# Does explaining the origins of misinformation improve the effectiveness of a given correction?

**DOI:** 10.3758/s13421-022-01354-7

**Published:** 2022-09-20

**Authors:** Saoirse Connor Desai, Stian Reimers

**Affiliations:** 1 School of Psychology, University of New South Wales, 1006, Mathews Building, 8, Kensington, NSW 2052 USA; 2 Department of Psychology City, University of London, London, UK

**Keywords:** Misinformation, Continued influence effect, Explanation, Correction

## Abstract

Misinformation often has a continuing influence on event-related reasoning even when it is clearly and credibly corrected; this is referred to as the *continued influence effect*. The present work investigated whether a correction’s effectiveness can be improved by explaining the origins of the misinformation. In two experiments, we examined whether a correction that explained misinformation as originating either from intentional deception or an unintentional error was more effective than a correction that only identified the misinformation as false. Experiment 2 found no evidence that corrections explaining the reason the misinformation was presented, were more effective than a correction not accompanied by an explanation, and no evidence of a difference in effectiveness between a correction that explained the misinformation as intentional deception and one that explained it as unintentional error. We replicated this in Experiment 2 and found substantial attenuation of the continued influence effect in a novel scenario with the same underlying structure. Overall, the results suggest that informing people of the cause leading to presentation of misinformation, whether deliberate or accidental, may not be an effective correction strategy over and above stating that the misinformation is false.

People are often faced with information they subsequently learn is false. Incomplete, incorrect, and inaccurate reports can circulate through social media and journalistic channels, before eventually being corrected. Even when misinformation is swiftly and credibly corrected, many studies have shown that misinformation often has a continuing influence on memory and reasoning; this is known as the continued influence effect of misinformation (CIE; Johnson & Seifert, 1994; Chan & et al. 2017; Sanderson, Ecker, & Sanderson, 2020; Lewandowsky, Ecker, Seifert, & et al. 2012; Ecker, Lewandowsky, Swire, & et al. 2011; Walter & Murphy, 2018; Walter & Tukachinsky, 2020; Ecker, O’Reilly, & et al. 2020; Ecker & Antonio, 2021; Ecker & Ang, 2019). The harmful consequences of misinformation for society make establishing effective methods of correction particularly important (Lewandowsky, Ecker, & Cook, 2017).

The effectiveness of corrections to misinformation may depend on the reason the misinformation was originally disseminated. Misinformation can be disseminated intentionally or unintentionally (Lewandowsky, Cook, & et al. 2020; Kozyreva, Lewandowsky, & Hertwig, 2020). One example of misinformation that was spread unintentionally occurred at a news conference in 2021, when the Chief Scientific Advisor to the UK government mistakenly stated that 60% of people admitted to hospital with COVID-19 in England had been fully vaccinated. Despite later correcting the error on social media, the initial statement had already circulated widely and was cited as evidence that COVID-19 vaccines are ineffective (Asenso, 2021). Misinformation can also be entirely fabricated and spread with the intention to mislead or deceive (Lewandowsky, Stritzke, Freund, & et al. 2013; Green, 2018; Lewandowsky et al., 2012; Lewandowsky et al., 2020). The present study examined whether explaining that misinformation originated from a lie or an accidental error can improve a correction’s effectiveness over a correction that merely identifies the information as false.

## The continued influence effect

The CIE is typically measured using a fictional scenario paradigm (see Wilkes & Leatherbarrow 1988; Johnson & Seifert, 1994). In a standard CIE task, participants read a report of an unfolding event (e.g., a warehouse fire) that includes causal information (e.g., “the fire was caused by carelessly stored flammable liquids”). This causal information is either presented and subsequently corrected (e.g., “flammable liquids did not cause the fire”), remains uncorrected, or is never presented at all (Johnson & Seifert, 1994; Wilkes & Leatherbarrow, 1988; Connor Desai & Reimers, 2019; Guillory & Geraci, 2010; Hardwicke, 2016; Bush, Johnson, & Seifert, 1994). Participants’ responses to later inference questions (e.g., “what could have caused the explosions?”) typically show that the corrected cause (i.e., “there were flammable chemicals in the closet”) has a continued influence on event-related reasoning, despite memory for event-related details remaining intact^1^.

Although a correction typically decreases reliance on misinformation relative to a *no correction* condition, it often fails to reduce reliance on misinformation to the level of a *no misinformation* baseline (Johnson & Seifert, 1994; Ecker, Lewandowsky, & Tang, 2010; Ecker et al., 2011; Ecker, Lewandowsky, & Apai, 2011). The CIE has been observed with different types of misinformation; for instance, with false beliefs about education (Ferrero, Konstantinidis, & Vadillo, 2020; Ferrero, Hardwicke, & et al. 2020), political misinformation (Swire, Berinsky, & et al. 2017; Ecker & Ang, 2019; Guillory, 2016; Nyhan & Reifler, 2015; Gordon, Ecker, & Lewandowsky, 2019), commonly believed myths (e.g., *liars give themselves away with physical tells*; Swire, Ecker, and Lewandowsky (2017)), as well as misinformation in newspaper headlines (Ecker, Lewandowsky, Chang, & et al. 2014).

The CIE has primarily been explained in terms of memory-updating and retrieval processes. The selective retrieval account holds that the CIE occurs when the misinformation is successfully retrieved but the correction is not (Ecker, Hogan, & Lewandowsky, 2017; Ecker et al., 2010; Lewandowsky et al., 2012; Ecker et al., 2011; Ecker, Swire, & Lewandowsky, 2014; Ecker et al., 2011). The model-updating account alternatively argues that the CIE is driven by a failure to integrate the updated information into a mental model of the described event constructed around the misinformation, unless an alternative explanation is available (Johnson & Seifert, 1994; Gordon, Brooks, & et al. 2017; Brydges, Gordon, & Ecker, 2020; Kendeou & et al. 2014; Wilkes & Leatherbarrow, 1988). The selective retrieval account implies the misinformation can be retrieved without the correction whereas the model-updating account suggests that although the specific information can be corrected, the mental model cannot.

### Explaining the origin of the misinformation

Both selective retrieval and model-updating accounts treat the CIE as a memory bias in which, either a correction is not incorporated into one’s mental model of a described event or is not successfully retrieved. Both accounts would predict that a more salient correction such as a correction that explains the origins of the misinformation to be more effective in reducing post-correction reliance on misinformation. More detailed corrective information (i.e., one that explains why the misinformation is incorrect) might encourage detection of inconsistencies between the misinformation and correction (Swire et al., 2017; Guzzetti, 2000; Kendeou & et al. 2014). Either way, a more salient correction to the misinformation should enable more successful model updating or retrieval of the correction information.

Explaining the origins of the misinformation may also influence the pragmatic inferences that people can make about the correction of misinformation (Lewandowsky et al., 2012; Grice, 1975; Seifert, 2002). People might make inferences about the reasons the original misinformation was presented, the intentions of the actors involved in the story, and the relative reliability of initial misinformation and correction (e.g., Connor Desai, Pilditch, & Madsen, 2020; Pilditch, Madsen, & Custers, 2020; Pilditch, Fries, & Lagnado, 2019). From a conversational perspective, corrections that do not explain the origin of the misinformation should be challenging when interpreting written or spoken statements, if the correction to misinformation only addresses the literal content of misinformation (e.g., there were no flammable chemicals on the premises), but not the conversational implications of misinformation (i.e., why the misinformation was reported in the first place; Bush et al., 1994; Johnson & Seifert, 1994; Seifert, 2002; Sperber et al., 2010). For example, in the warehouse fire scenario described earlier, the correction (i.e., “that flammable liquids initially thought to be in the warehouse were never actually there”), states that the original statement was false, without explaining how or why the misinformation was initially presented. Without any explanation, participants might be unsure whether the correction is any more valid than the original misinformation, and consequently, still give weight to the first piece of information they encountered (Connor Desai et al., 2020).

To date, there has been little research examining whether corrections that explain the origins of the misinformation (such as from a deliberate lie or a mistake) are more effective than corrections that merely label the misinformation as false. To the best of our knowledge, only one previous CIE study has directly examined the effectiveness of corrections that explain how the misinformation originated. In their study, Bush et al. (1994) examined whether corrections that explain the misinformation in terms of the communicative intentions behind the misinformation were more effective than those that explain the literal content of the misinformation. They presented participants with the warehouse fire scenario described earlier and examined the effectiveness of two explanatory corrections; the correction either explained why the misinformation may have been presented initially, but was irrelevant in the current context (“*the expected delivery of paint and gas cylinders had not arrived*”), or explained how the misinformation may have been presented in error (“*the closet actually had coffee and soda cans rather than paint and gas cylinders*”). There was a marginal difference between the effectiveness of explanatory and non-explanatory corrections. In the present study, we provided a more salient explanation by explicitly mentioning the source of the misinformation and attributing the misinformation either to deliberate attempt at deception or to an individual’s genuine mistake.

### Sources of misinformation: Lies vs. errors

Explaining the origins of the misinformation may generally enhance a correction’s effectiveness, but corrections that appeal to different sources of misinformation may differ in their effectiveness (Lewandowsky et al., 2012). Although there are many sources of misinformation, two are dominant: lies and errors. Misinformation can occur due to simple errors such as insufficient fact-checking, hasty reporting, or from misunderstanding of event-related details. Misinformation can also arise from deception. For instance, when sources lie, or informants have a vested interest in cultivating belief in an alternative version of events (Green, 2018). Although both forms can lead to presentation of the same misinformation, they may have different effects on people’s inferences. While errors focus on the causal sequence of events deception involves considering the actors’ motivations. The distinction between lies and errors has similarities to research in other areas of psychology examining the differences between inadvertent and intentional morally transgressive behavior (e.g., Cushman 2008; Young & Saxe, 2009).

Previous studies suggest that people might be more likely to discount information from a deceptive source than one who made a genuine error. For instance, people discount eyewitness testimony when they are told that the eyewitness had a longstanding grudge against the suspect (Lagnado & Harvey, 2008), and are more likely to discount an intentionally deceptive alibi than a mistaken alibi (Lagnado, Fenton, & Neil, 2013). There is also evidence to suggest that people perceive intentional actions as more “causal” and blameworthy than unintentional actions (Lagnado & Channon, 2008). Furthermore, people are also less susceptible to the deleterious effects of misinformation (Lewandowsky, Stritzke, Oberauer, & et al. 2005), and inadmissible evidence (Fein, McCloskey, & Tomlinson, 1997), when they are given reason to be suspicious of the motives behind its introduction.

Other studies have found that the type of explanation provided for discounting false information does not make a difference. In their study, Green and Donahue (2011) found that participants did not correct their beliefs about a report irrespective of whether they were subsequently informed that the author of the report had “made it up” or that the report was inaccurate because of a “mix-up”. Participants derogated a lying author’s character more than an author who made an error, but misinformation continued to influence story-related beliefs equally whether the story contained inaccuracies due to a genuine error or intentional deception. Overall, although the findings are somewhat mixed, they suggest that informing people that an informant was intentionally deceptive might be more effective than explaining that the misinformation originated from an error.

### Overview of experiments

The aim of the research presented here was twofold. Our chief aim was to examine whether corrections that explain the origins of the misinformation are more effective than corrections that provide no such explanation. Providing an explanation for the origins of the misinformation could facilitate correction processing (Johnson & Seifert, 1994; Gordon et al., 2017; Lewandowsky et al., 2012; Brydges et al., 2020), or enhance later retrieval of the correction (Ecker et al., 2011; Swire et al., 2017; Gordon et al., 2017). Alternatively, an explanation may be insufficient for individuals to reconsider the story and correct for inaccuracies (e.g., Green & Donahue, 2011).

The second aim was to compare the relative effectiveness of explanations involving deception versus those involving errors in reducing the CIE. The discussion above suggests that it is possible that a correction explaining the misinformation as a deliberate lie may be more effective than one explaining it as an error. As such, we presented participants with simple corrections, or with corrections which explained either that the misinformation was an accidental error or a deliberate lie. Experiment 2 tested the impact of explanatory corrections using the warehouse fire scenario. Experiment 2 replicated this and extended it to compare the effectiveness different types of correction in a novel scenario describing a van crash.

## Experiment 1

Experiment 1 tested whether corrections that explain a piece of misinformation as originating from a deliberate lie or a genuine error are more effective than a correction that simply states misinformation is false. We tested this in the warehouse fire scenario described earlier but presented this as a series of social media posts. We predicted that a correction would reduce the number of references to misinformation compared to *no correction*, and that there would be fewer misinformation references following a correction that explains how the misinformation originated (i.e., lie or an error) than a correction that does not explain the misinformation’s origins. We made no strong prediction on the relative impact of lie or error-based explanations but examined whether there is evidence deception being more effective than error in correcting misinformation.

### Participants

Three-hundred and sixty-five U.S.-based participants were recruited via Amazon Mechanical Turk (MTurk) to retain at least 70 participants per condition. Only participants with a human intelligence task (HIT) approval rating greater than, or equal to 99%, were recruited for the experiment. Participants had a mean age of 39.38 (*SD* = 11.92), and there were 169 females and 196 males. Participants were paid $1.50 for their time (*Mdn* = 16 min).

### Design and materials

**Fig. 1:**
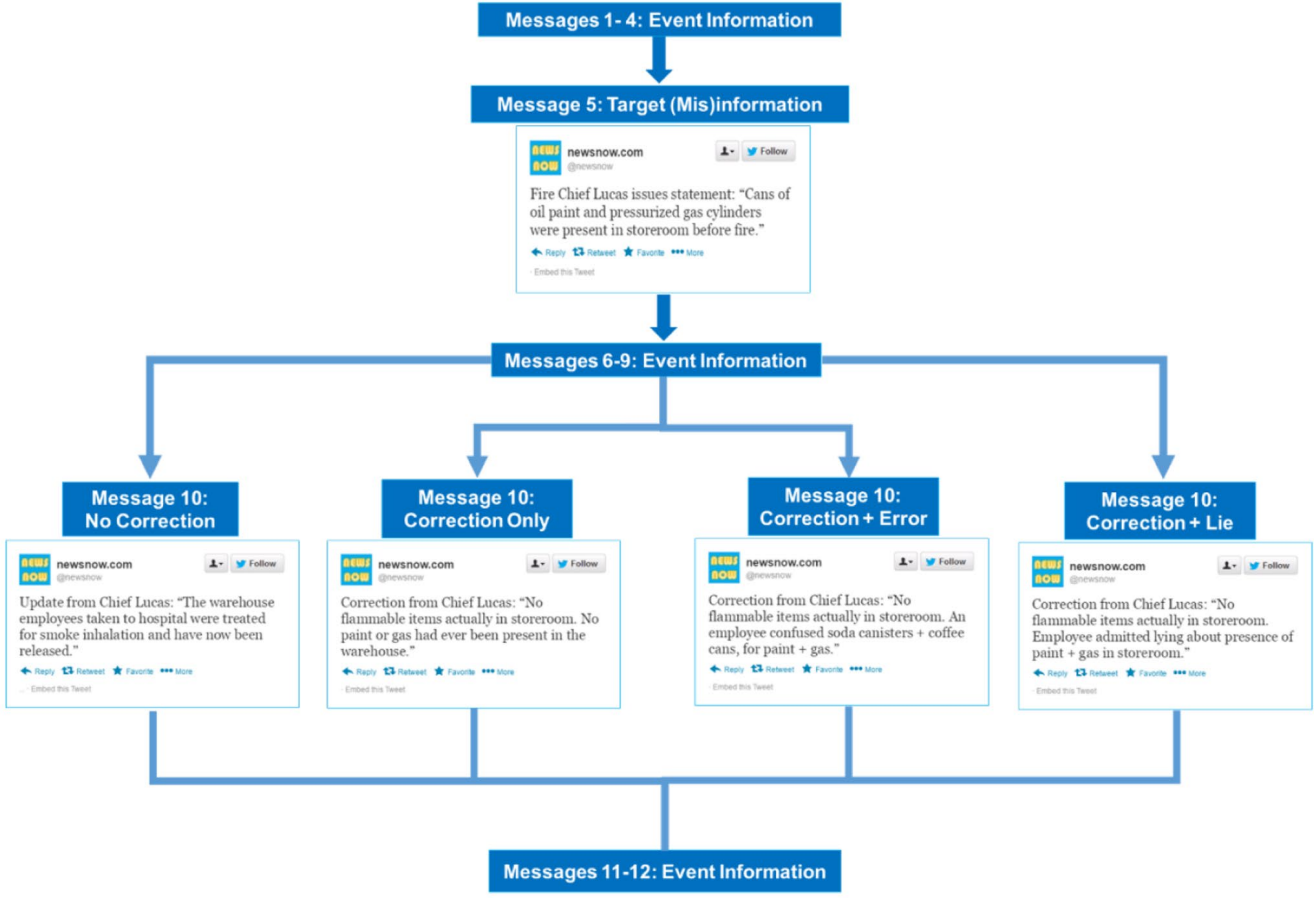
Schematic diagram of information presented in Experiment 2

We randomly assigned participants to one of four between-subjects conditions: the *no correction*, *correction, correction + error explanation*, or *correction + lie explanation* groups (see Fig. 1). There were two primary measures: open-ended questions that required participants to make inferences about the scenario and questions on the scenario’s factual details. We also asked the participants two questions assessing participant’s awareness of the correction. The primary dependent variable was the mean number of references to misinformation in response to the open-ended inference questionnaire.

Participants read one of four versions of a fictional news report about a fire at a stationery warehouse, each consisting of 12 sequentially presented statements. The warehouse fire scenario has been used in several earlier continued influence effect studies (Connor Desai & Reimers, 2019; Guillory & Geraci, 2010; Johnson & Seifert, 1994; Wilkes & Leatherbarrow, 1988). We made two key changes to the warehouse fire scenario in the present study. First, we modified the news report’s presentation format, which we presented as a series of Tweets (cf. Hardwicke 2016), to resemble the appearance of breaking news stories on Twitter. The Tweets originated from the same fictional news outlet, called “News Now”, and did not exceed 140 characters (see Fig. 1). Second, we modified the peripheral story details (i.e., details other than the misinformation or correction), such that they were relatively neutral with respect to the misinformation.^2^ In all other respects, the scenario was the same use used in previous studies.

### Procedure

Participants clicked on a link in MTurk to enter the experimental site. They subsequently read details about the experiment, gave consent, and completed an instructional manipulation check (IMC). The IMC involved participants reading a paragraph explaining that experimental manipulations are ineffective if participants do not read the instructions properly. The paragraph concludes by asking participants to demonstrate that they have read the instructions, by ignoring the check-boxes that appear below the paragraph and click “continue” (Oppenheimer, Meyvis, & Davidenko, 2009). Participants (*N* = 4) who did not read the instructions accurately were not permitted to complete the study. There were no other exclusion/inclusion criteria.

The remaining participants received instructions that the study explored the factors that affect people’s judgments about news reports: to read a brief report about an investigation into a fire, complete a short questionnaire about the report, and provide demographic information. Participants were informed that they would not be able to backtrack and that each message would appear for a minimum of 5 s before they could move on to the next message. Participants then read one of the four condition-dependent versions of the warehouse fire scenario.

After reading the report, participants completed a questionnaire about the scenario: seven inference questions, seven factual recall questions, and two questions probing awareness of the correction information. Inference and factual recall question blocks were intermixed and presented in random order, except the question probing the fire’s most likely cause, which always came last. Participants typed a response to each of the 16 questions in a text box, they were required to use a minimum of 25 characters, and encouraged to answer using full sentences. After completing the questionnaire, participants provided their sex, age, and their highest level of education.

## Results

We used Bayesian regression to analyze participants’ responses because it allowed us to examine degrees of credibility rather than dichotomous indicators of significance or non-significance. All results were determined using the brms package in R (Bürkner, 2017). We report 95% highest posterior density (HPD) intervals for planned contrasts on model parameters. There is no evidence for a difference between groups if the 95% HPD interval includes the null value of one^3^.

### Coding of responses

We used responses to three types of questions in the analysis: Inference questions in which participants speculated about the cause of the fire; factual recall questions to assess engagement with and understanding of the information they received; awareness of correction questions explicitly asking participants if they remembered any information being corrected in the report. Participants answered the seven inference questions based on their understanding of the report. Responses to inference questions were coded as a reference to misinformation if they explicitly stated or strongly implied that oil paint and gas cylinders caused or contributed to the fire and were scored zero otherwise (examples of responses scored one and zero for each inference question can be found in Table 5 of the Appendix).

Participants could answer the factual recall questions by recalling the details of the report. Each response was coded as one when the participant fully or partially recalled the detail correctly and scored zero if it was not. For example, in response to the question *“Where was the warehouse located?”* a full recall response would be *“Fern Hill Industrial Park”* but any responses that recalled some of these details were also scored one (e.g., *“Fern Hill”*, *“industrial park”*, *“industrial area”*)^4^. There was a minimum recall accuracy score of zero and a maximum score of seven. We computed awareness of correction scores using the same criteria; the maximum individual awareness of correction score was two.

#### Inter-coder reliability

All responses were coded by a scorer who was naive to the experimental conditions using a standardized scoring guide. A second, independent coder received instructions on the coding scheme and coded 10% of participants’ responses (*n* = 36). Inter-rater agreement was 0.88 and Cohen’s *κ* = 0.76 ± 0.03, indicating a high level of agreement between coders, both of which are higher than the benchmark values of 0.7 and 0.6 (Landis & Koch, 1977). When coders disagreed, we relied on the first coder’s ratings.

### Inference scores

**Fig. 2:**
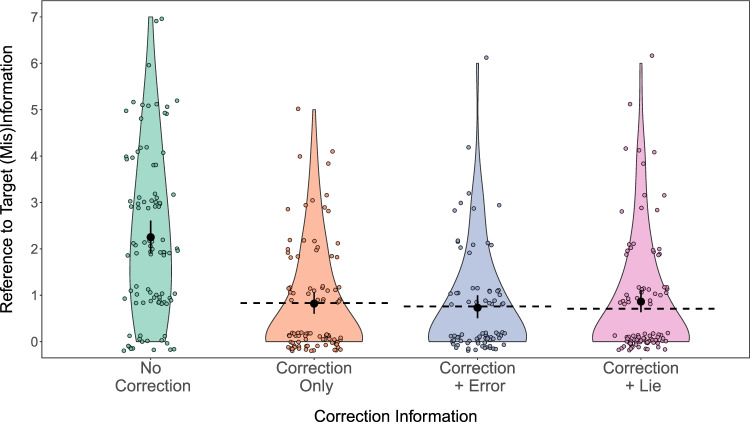
Violin plots show the distribution and probability density of references to misinformation by correction information condition in Experiment 2. The violin plot is a symmetrical rotated kernel density plot and shows the density of the data at different values. *Black points* represent mean and 95% confidence interval of the mean. *Dashed lines* represent condition means after excluding participants who did not recall the correction. *Colored dots* show individual data points

Figure 2 shows that relative to the *no correction* group, there were fewer references to misinformation in the correction groups. We fit a Bayesian negative binomial model to inference scores (i.e., the number of references to misinformation) with correction condition (no correction, correction only, correction + error, correction + lie) as a fixed predictor. We obtained 95% highest posterior density intervals to examine the evidence that: 1) correction conditions differed from a *no correction* condition, and 2) the three correction conditions differed from each other. Table 1 shows that all 95% HPD intervals comparing the *no correction* group to the three correction groups did not include the null value of one. In contrast, all 95% HPD intervals for contrasts between the three correction groups did include the null value. Overall, there was evidence for a difference between the *no correction* group and correction groups, but no evidence for a difference between correction groups.

**Table 1:** Planned contrasts on inference scores in Experiment 2

Contrast	Ratio	Lower HPD	Upper HPD
No Correction - Correction + Error	3.309	2.155	4.59
No Correction - Correction + Lie	2.838	1.951	3.83
No Correction - Correction Only	2.942	2.063	4.11
Correction + Error - Correction + Lie	0.858	0.560	1.28
Correction + Error - Correction Only	0.890	0.562	1.32
Correction + Lie - Correction Only	1.045	0.662	1.47

#### Correction acknowledgment

A key claim from the CIE literature is that people often continue to rely on misinformation despite clearly understanding and recalling that the misinformation was corrected (Johnson & Seifert, 1994). We tested this by calculating the proportion of participants who correctly recalled the correction and referred to misinformation in response to at least one inference question. A substantial minority of participants in each correction condition made at least one reference to misinformation on inference questions while acknowledging the correction: 27% of the *correction only* group, 23% of the *correction + error* group, and 28% of the *correction + lie* group. A considerable number of participants (36-44%) showed no *continued influence effect*; that is, they accurately recalled the correction and made no references to misinformation. The remaining participants (13-20%) did not recall the correction information. Overall, we observed that a substantial proportion of participants who received a correction continued to refer to misinformation despite acknowledging the correction.

### Recall accuracy scores

We fit a Bayesian binomial regression model to recall accuracy scores to examine whether there was evidence for differences between the groups. Table 2 shows that all 95% HPD intervals included the null value of one. Overall, there was no evidence for a difference in recall scores between groups.

**Table 2:** Contrasts for recall accuracy scores in Experiment 2

Contrast	Odds ratio	Lower HPD	Upper HPD
No Correction - Correction + Error	0.891	0.744	1.04
No Correction - Correction + Lie	0.945	0.798	1.09
No Correction - Correction Only	0.923	0.783	1.07
Correction + Error - Correction + Lie	1.058	0.899	1.24
Correction + Error - Correction Only	1.035	0.878	1.21
Correction + Lie - Correction Only	0.977	0.830	1.13

## Discussion

This experiment examined whether corrections which explain the origins of the misinformation are more effective than corrections which simply identify the misinformation. We found no evidence for differences among the three correction groups: simple correction without explanation, unintentional error, and intentional deception. Almost a third of participants in each of the three correction groups made at least one reference to misinformation and exhibited a CIE. That is, they acknowledged the correction and causally referred to the misinformation. Experiment 2’s results therefore suggest that there is no additional benefit gained by explaining that misinformation originated from either a lie or an error, and no differences between lie- and error-based explanations in reducing the CIE.

To corroborate this finding, in Experiment 2 we attempted to replicate the findings of Experiment 2 and generalize the findings to a novel scenario. Establishing whether an experimental effect is present with a single stimulus scenario can limit the scope of the conclusions reached. Including multiple scenario versions can therefore increase confidence that the results generalize across scenarios (Monin & Oppenheimer, 2014; Westfall, Judd, & Kenny, 2015). Accordingly, Experiment 2 compared inferences from the warehouse fire scenario to a scenario with the same underlying structure, but a different subject matter, to examine whether the null effect of explanatory corrections extended to other scenarios.

## Experiment 2

Experiment 2 explored the effectiveness of corrections that explain the origins of the misinformation in the warehouse fire scenario and a new scenario describing a van crash. The scenarios in Experiment 2 also included a statement describing other potential causes of the outcome described in the scenario (“report from the fire department indicates most industrial fires are due to equipment and machinery, flammable substances, hot work, and electrical hazards”). We included this statement so that participants had alternative explanations available in memory to answer inferential questions even though the initial cause (i.e., misinformation) had been corrected, and to avoid “don’t know” responses^5^.

### Participants

A power analysis based on an effect size from a pilot study indicated that a minimum of 110 participants (*f* = 0.40, 1-β = 0.95, *α* = 0.05) would be required in order to detect a main effect of correction information (*df* = 3, *k* = 8). One hundred and sixty-three participants completed the experiment via MTurk with the intention to retain 20 participants per condition. Four participants were excluded before analysis because they failed a recognition test of the correction/control message. Another participant wrote nonsensical responses, so their responses were excluded from the analysis. We included 158 participants in the final analysis. Participants had a mean age of 39.62 (*SD* = 11.21), and there were 69 females and 89 males. Participants were paid $1.50 for their time (*Mdn* = 16 min).

### Design and materials

**Fig. 3:**
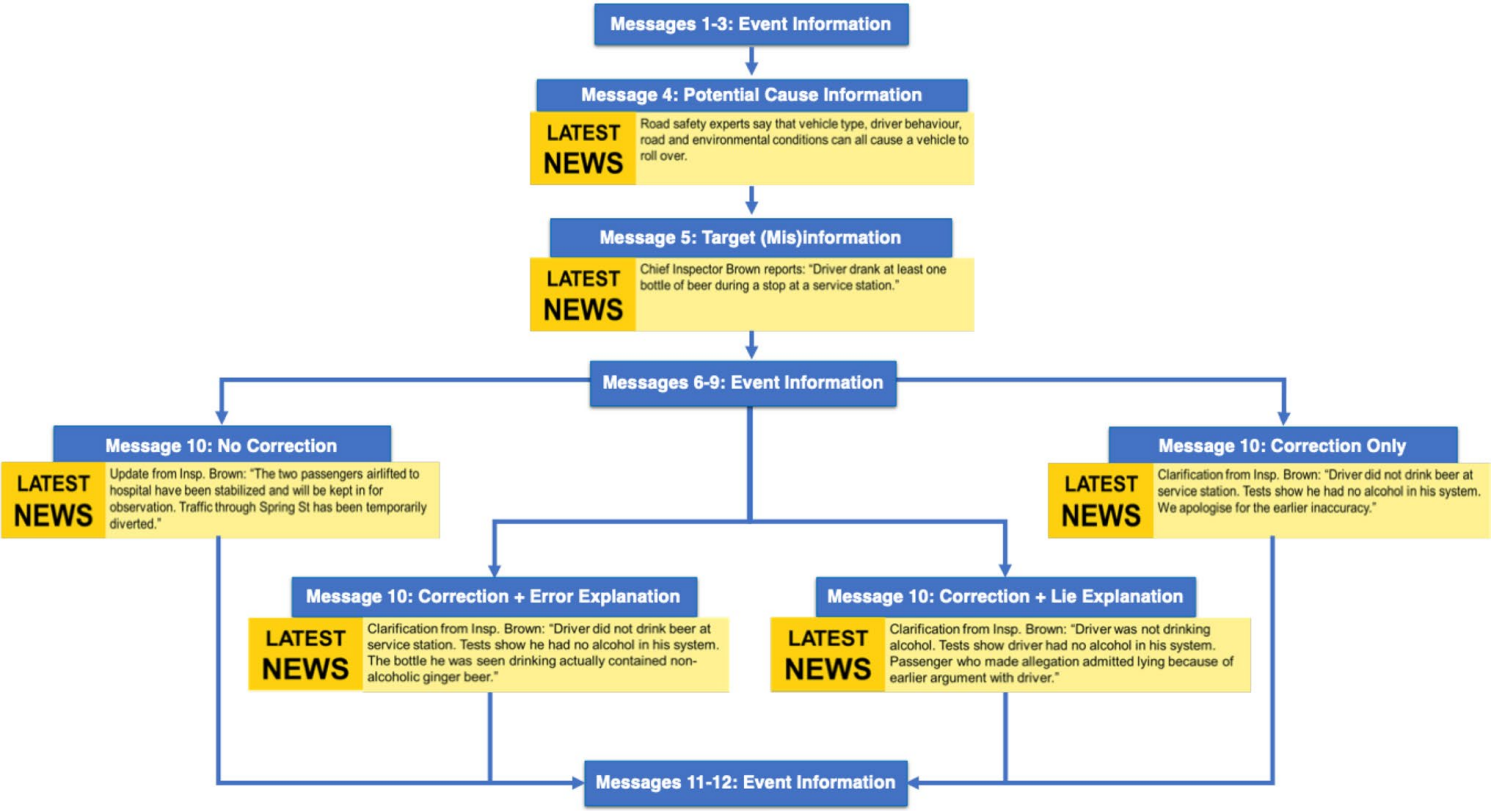
Schematic diagram of Experiment 2

A 2 (Scenario: Van Crash, Warehouse Fire) x 4 (Correction Information: No Correction, Correction Only, Correction + Error, Correction + Lie) between-subjects factorial design was used such that there were four versions of the warehouse fire and the van crash scenario (see Fig. 3). There was a random allocation of participants to one of the eight experimental conditions.

The materials consisted of two different scenarios (warehouse fire, van crash) presented in individual breaking news statements originating from the same fictional news source. The maximum character length per message increased from 140 to 280 to allow for additional information in the explanatory correction messages. Messages were approximately matched for the number of characters and words across experimental conditions.

The new scenario described a van that had crashed while returning from a music festival. The misinformation suggested that the van had crashed because the driver had been drinking. To directly compare between scenarios, we modeled the inference questions for the van crash scenario on those used for the warehouse fire scenario. For example, an inference question for the warehouse fire scenario asked, *“How could the fire at the warehouse have been avoided?”* and for the van crash scenario, similarly asked, *“How could this accident have been avoided?”*.

### Procedure

All elements of the experimental procedure were identical to those of Experiment 2, except as stated below. We changed the instruction check and added a recognition test examining whether participants had encoded the correction. The instruction check appeared after reading instructions and before beginning the experiment. Participants answered three questions about the main instructions (e.g., *what is the minimum time each statement will appear on the screen?*). We included this instruction check to encourage participants’ attentiveness throughout the experiment, but there was no consequence of failing the test. The instruction check was updated to be consistent with developing best practice in the field (Berinsky, Margolis, & Sances, 2014; Hauser & Schwarz, 2016; Berinsky, 2016).

## Results

### Coding of responses

Responses which explicitly stated or strongly implied that the target misinformation was causally involved in the event were scored one and otherwise scored zero (e.g., *“the van crashed because the driver was drunk”*). Example responses to inference questions for the van crash scenario can be found in Table 6 of the Appendix. In the van crash scenario, references to driver behavior that did not mention intoxication or drunkenness with reference to the van crashing were not counted as references to misinformation (e.g., *“by having him be more alert drinking coffee”*). The maximum individual inference score was seven. We applied the same coding criteria from Experiment 2 to factual recall and awareness of correction responses. An example of a partial recall response for factual recall questions in the van scenario was in response to the question “What event was the van transporting people from?” the full correct answer would be “Beat bunker musical festival”. We accepted as correct “music festival”, “concert” or anything any response that captured the van was transporting people back from a live music event.

#### Inter-coder reliability

A coder who was naive to the experimental conditions scored all responses. A second, independent judge then coded approximately 10% of participants’ responses. Inter-rater agreement was 0.90 and Cohen’s *κ* = 0.81 ± 0.05, indicating a very high level of agreement between coders.

### Inference scores

**Fig. 4:**
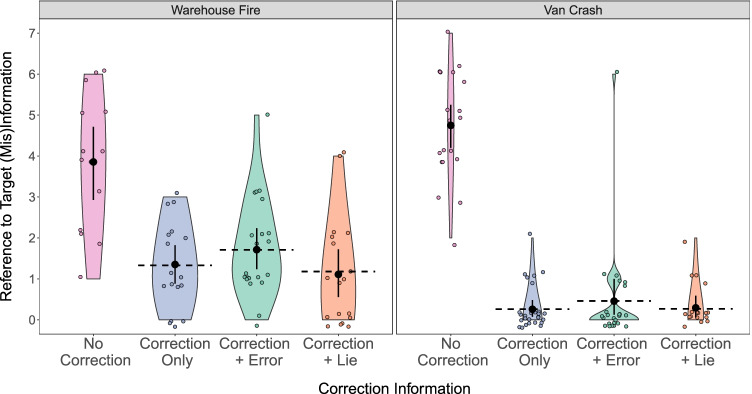
Violin plots show the distribution and probability density of references to misinformation by correction information condition and scenario in Experiment 2. The *black points* represent mean and 95% confidence interval of the mean, and the *dashed lines* represent condition means after excluding participants who did not recall the correction. *Colored dots* show individual data points

Figure 4 shows the number of references to misinformation as a function of scenario and correction group. Table 3 shows contrasts performed on Bayesian negative binomial regression parameters. Consistent with Experiment 2, the relative number of references to misinformation were higher in the *no correction* and correction groups and there was evidence for a difference between groups as the 95% HPD intervals did not include the null value. There was no evidence for the difference between the three different correction groups, however.

**Table 3:** Contrasts for inference scores in Experiment 2

Contrast		Ratio	Lower HPD	Upper HPD
Warehouse fire	No Correction - Correction + Error	2.241	1.351	3.38
	No Correction - Correction + Lie	3.469	1.846	5.66
	No Correction - Correction Only	2.834	1.581	4.54
	Correction + Error - Correction + Lie	1.542	0.560	2.56
	Correction + Error - Correction Only	1.259	0.562	2.05
	Correction + Lie - Correction Only	0.818	0.662	1.38
Van Crash	No Correction - Correction + Error	10.768	5.388	19.64
	No Correction - Correction + Lie	17.40	5.986	45.01
	No Correction - Correction Only	18.969	8.391	41.99
	Correction + Error - Correction + Lie	1.619	0.373	4.49
	Correction + Error - Correction Only	1.788	0.474	4.07
	Correction + Lie - Correction Only	1.112	0.167	2.98

There was a similar pattern of results for the van crash scenario. However, the difference between the number of references to misinformation between the *no correction* and correction groups was larger than for the warehouse fire scenario. There was, as before, no evidence for any difference between the three different types of correction.

#### Correction acknowledgement

In the warehouse fire scenario, 81% of participants in the *correction + error* group referred to misinformation at least once and acknowledged the correction; this was 50% in the *correction + lie* group, and 65% in the *correction only* group. Between 5 and 33% of participants across conditions who read the warehouse fire scenario showed no continued influence effect; they accurately recalled the correction and made no references to the misinformation. The remaining 5-12% of participants across conditions did not recall the correction when probed.

In contrast, in the van crash scenario, only 25% of participants in the *correction + error* group; 18% of the *correction + lie* condition; and 22% of the *correction only* group referred to the misinformation at least once while also acknowledging that it had been corrected. Most participants (65-77%) showed no continued influence effect and accurately recalled the correction. Between 0 and 12% of participants who read the van crash scenario did not recall the correction. Notably, more than twice the number of participants referred to misinformation and acknowledged the correction after reading the warehouse fire scenario than the van crash scenario.

### Recall accuracy scores

We examined whether there was evidence that the correction manipulation influenced recall accuracy. Table 4 shows contrasts performed on the Bayesian binomial regression model parameters. All 95% HPD intervals included the null value of one indicating no evidence of a difference between correction groups in either the warehouse fire or van crash scenarios.

**Table 4:** Planned contrasts on recall accuracy scores in Experiment 2

Contrast		Odds ratio	Lower HPD	Upper HPD
Warehouse fire	No Correction - Correction + Error	0.817	0.564	1.14
	No Correction - Correction + Lie	0.822	0.564	1.14
	No Correction - Correction Only	1.100	0.687	1.55
	Correction + Error - Correction + Lie	1.005	0.693	1.32
	Correction + Error - Correction Only	1.351	0.952	1.86
	Correction + Lie - Correction Only	1.341	0.899	1.86
Van crash	No Correction - Correction + Error	1.049	0.744	1.36
	No Correction - Correction + Lie	1.073	0.756	1.46
	No Correction - Correction Only	0.988	0.725	1.30
	Correction + Error - Correction + Lie	1.025	0.711	1.36
	Correction + Error - Correction Only	0.945	0.699	1.22
	Correction + Lie - Correction Only	0.920	0.662	1.23

### Discussion

Experiment 2 again found no evidence that corrections which explain the origins of the misinformation were more effective as a simple correction. Results also indicated the mutability of the CIE - in a new scenario with the same structure as the warehouse fire scenario, far fewer participants referred to misinformation while acknowledging that the information was corrected when compared to the warehouse fire scenario. Experiment 2’s results provide further confirmation that corrections which explain how the misinformation originated (either from deliberate deceit or an accidental error) are no more effective than corrections that simply label the misinformation as false, and that there was no difference in efficacy between intentional deception and unintentional error.

## General discussion

The present research examined whether explaining that misinformation originated from an intentional deception or from an unintentional error can improve a correction’s effectiveness relative to a correction that merely labels the misinformation as false. In two experiments, we found no evidence that explaining the origins of the misinformation was a more effective correction strategy than a simple correction. Furthermore, there was no evidence of a difference between explaining that misinformation originated from a lie or an error.

These results suggest that informing people that misinformation originated from either a lie or an error may not be an effective correction strategy. Our findings are consistent with previous studies showing that corrections that accounted for the conversational implications of the misinformation (e.g., “X, which had originally been believed because of Y, is actually untrue”) somewhat reduce, but do not eliminate the CIE (Bush et al., 1994). The results are also consistent with work showing that people continue to believe stories after learning that the story contained inaccuracies due to accidental error or deliberate deception (Green & Donahue, 2011).

Consistent with previous studies on the CIE, all three types of correction reduced reliance on misinformation relative to the *no correction* control condition (e.g., Ecker et al., 2017; Ecker et al., 2011; Ecker et al., 2011). However, around a third of participants still referred to the misinformation even though it was corrected. We hesitate to say that corrections failed to eliminate the CIE, because, unlike some previous studies on the CIE, we did not include a “no misinformation” control condition meaning there is no baseline unprompted rate of references to the corrected cause with which to compare^6^. We do note that our response coding framework made it unlikely that the responses counted as references to misinformation would be made spontaneously without the misinformation being mentioned.

Both the model-updating (O’Rear & Radvansky, 2020; Gordon et al., 2017; Brydges et al., 2020; Johnson & Seifert, 1994; Rich & Zaragoza, 2016), and selective retrieval (Gordon et al., 2017; Ecker et al., 2011; Swire et al., 2017) accounts, suggest that more detailed corrections that provide an explanation for why the misinformation is incorrect should be more effective (Swire et al., 2017). According to this view, explaining that the misinformation originated from a genuine mistake or from willful deception should increase the correction’s salience and encourage more elaborate processing, making it more likely to be integrated during encoding, or successfully retrieved later. Our results do not appear to fit with either a model-updating or selective retrieval account of the CIE. The warehouse fire scenario explanations did not facilitate updating or boost retrieval of the correction in the way that more detailed and elaborate explanations for why the misinformation is incorrect do (Swire et al., 2017; Kendeou et al., 2014). One interpretation of the present results is that the explanation offered for how the misinformation originated was not detailed enough or did not sufficiently explain its origins; and consequently, did not reduce the CIE further than a correction without an explanation. However, we also found that all three types of correction were considerably more effective at reducing references to misinformation for the van crash than the warehouse fire scenario.

Another reason that we did not find evidence for a difference between corrections could be that people disregarded the explanation and focused on the negation (i.e., *that there were no flammable substances in the storeroom*) because the person who initially conveyed the misinformation was unreliable. Some courtroom simulation studies suggest that people are more convinced by physical evidence than eyewitness evidence (e.g., Skolnick & Shaw 2001), perhaps because people assume that human measuring devices (e.g., eyewitnesses) are inherently less reliable than physical ones (e.g., CCTV; see Lagnado et al., 2013). Explanatory corrections may be more effective if the correction involved a physical explanation of why misinformation is incorrect rather than a social explanation of a misunderstanding or deception.

Instilling a sense of distrust about the misinformation source’s motives may also be more effective at encoding than at retrieval (i.e., when misinformation is encoded rather than when the correction is presented). This explanation also fits with recent work showing that people are better able to incorporate information about constraints on an evidence sample when it is presented at encoding than when presented at retrieval (Ransom et al., 2022). However, we note that other studies have found this be an effective strategy at retrieval (Fein et al., 1997).

It may be the case for either or both types of explanation that the explanatory correction makes the original misinformation more salient, so where causal structure is lost, the simple misinformation (e.g., *“there was something about gas cylinders”*) might be more available. Whatever the causal explanation, it seems clear in these studies at least that there is no difference in efficacy between error- and lie-based corrections, since neither had any additional effect on CIE magnitude beyond a simple correction.

An important question that follows from the present findings is why a correction was more effective in the van crash than warehouse fire scenario. We matched the content, length, and serial position of the target (mis)information and correction statements, and peripheral details when constructing the scenarios. Given that the scenarios were structurally similar, the difference in effectiveness of corrections between scenarios could be due to the salience of individual statements, mapping onto existing representations in memory, or concreteness of the schema invoked by the scenario (e.g., Sadoski, Goetz, & Rodriguez, 2000; Anderson, 2018). Alternatively, people may be better able to process corrections to misinformation for scenarios where mechanisms by which misinformation is corrected are clearer (cf. Connor Desai, Xie, and Hayes (2022)). Future studies should examine the types of causal scenarios that give rise to the CIE to establish the boundary conditions of the effect.

Finally, in both experiments, the average number of references to misinformation was relatively low across all conditions. Across other studies that have used the continued influence paradigm, the frequency of references to corrected misinformation in similar conditions has varied substantially (cf. Ecker et al., 2011; Johnson & Seifert 1994). Here, although the number of references to misinformation in correction conditions was similar to that seen in correction conditions in some other studies, such as Ecker et al. (2011), it was lower than anticipated. Increasing the number of inference questions to give participants more opportunity to refer to the corrected misinformation might increase the experimental sensitivity to any differences between correction conditions.

### Errors vs. lies

We focused on the two major reasons why misinformation might occur: genuine mistake or deliberate deception. As discussed previously, we were interested in whether corrections that explained the misinformation as deception might be more effective in reducing the CIE than those that explained it as error. In both experiments, we did not find evidence for a difference between these two correction types, insofar as neither had any additional effect beyond a simple correction. In this case, there may be multiple effects that cancel each other out - for example, a deception-based correction might make a participant more convinced that the original information was false, but might also make them suspicious of the correction as well as the misinformation (i.e., if one person can lie, who’s to say the person correcting the information is telling the truth? e.g., Connor Desai et al., 2020). Similarly, a correction describing an error might make participants convinced that the original statement was incorrect but might also make them doubt the accuracy of other statements in the scenario.

Our primary goal was to examine whether the continued influence effect of misinformation could be more effectively reduced when corrected by informing participants that it originated from a lie or an error, than when no explanation for its origins is given. In Experiment 2, we found corrections with and without an explanation to be equally effective at reducing references to misinformation relative to no correction in the warehouse fire scenario. Furthermore, neither the elaborations explaining the cause of a mistaken belief nor those explaining a deliberate attempt at deception were more effective than a simple correction. In Experiment 2 we replicated this finding and examined whether it generalized to a new scenario involving a van crash. Results showed the CIE was substantially attenuated in the van crash scenario compared to the warehouse fire scenario. Given that the corrections in this scenario were almost entirely successful, it is unclear to what extent the effectiveness of corrections that explain the origins of the misinformation differs depending on the surrounding context. Although inter-scenario differences in the magnitude of the CIE were not the primary focus of our investigation, the discrepancy was unexpected.

## Conclusions

The current studies provide clear evidence that explaining the reason for the presentation of misinformation, whether the cause was an understandable mistake or dishonesty, does not necessarily reduce people’s reliance on misinformation in generating causal explanations for an event. These findings have crucial real-world implications, suggesting that providing explanations for why misinformation was originally reported might not be any more effective than simply issuing a correction. Further work will be needed to establish the generality of these findings, and perhaps uncover more nuanced ways in which broader context behind the initial presentation of misinformation can help reduce the CIE further. However, our findings add to the evidence that corrections explaining the source of misinformation are not necessarily more effective than simple corrections.

## Appendix

**Table 5:** Example response coding for inference questions in Experiment 2

Inference question	Response scored 1	Response scored 0
Is there any evidence of careless management in relation to this fire?	Yes, there were cans of oil paint and gas cylinders present in a storeroom before the fire.	It is unclear how this could have been avoided. Updated fire systems may have detected the blaze earlier, making it easier to control.
How could the fire at the warehouse have been avoided?	The fire at the warehouse could have been avoided by keeping accelerants and explosives such as pressurized gas and flammable paints in a designated contained area, per fire safety code	Whether or not the fire could have been avoided would depend on whether the facility was compliant with safety regulations during their inspection.
What precautions could be taken in the future to ensure this doesn’t happen again?	The oil paints and gas cylinders could be kept in a fire-safe vault of some sort, and certainly not within range of all the paper products at the warehouse.	Stricter and/or more surprise safety inspection would help.
What aspect of the fire should the police focus on in their investigation?	The police should focus on what ignited the paint/gas in the first place.	Lack of safety protocol being followed by management/employees being the potential cause.
Does any aspect of the fire deserve further investigation?	Other than investigating the reports among also finding out if it was caused by carelessness (like leaving oil paint cans and pressurized gas cylinders out of place), I would say there aren’t any.	No, not until the results of the fire inspection is reported.
Do you think any workers should be disciplined for their role in the fire?	I would say yes, if the oil paint and gas cylinders were not stored properly.	Yes, I think if the fire was started intentionally then the workers responsible should be disciplined.
What was the most likely cause of the fire?	The cause of the fire was most likely a rogue spark igniting either a pressurized gas canister or oil paint container.	Unsafe containers probably were likely.

**Table 6:** Example response coding for inference questions in van crash scenario

Inference question	Response scored 1	Response scored 0
What evidence is there of negligent driving in relation to this accident?	Yes, the driver had been drinking.	The van swerving off and toppling over could be a sign of negligent driving.
How could this accident have been avoided?	Paying closer attention, not drinking.	The accident could have been avoided if the driver was more cautious.
Were any of the people in the vehicle particularly responsible for the crash?	The driver who had alcohol in their system.	The driver was responsible for the crash.
What measures could the charter van company take to prevent future accidents?	Hire people who don’t drink on the job.	Inspect it more carefully, and make sure there is a limit on how many passengers are allowed in it at a time.
What aspects of the accident should further investigations be focused on?	The drinking of beer on the part of the driver.	Why the vehicle veered off the road.
For what reasons might the passengers want to take legal action against the charter van company?	Driver was drinking beer.	The passengers may pursue legal action against the company for medical costs, possible punitive damages if negligence is determined.
What do you think the most likely cause of the crash was?	Consumption of the beer by driver.	The behavior of the driver, road, and the type of the vehicle.

## Footnotes

1 CIE studies often measure reliance on misinformation by coding responses to open-ended questions. However, the effect has also been observed with direct measures of continued reliance on misinformation, such as belief in the misinformation (Guillory, 2013; Rich & Zaragoza, 2016), and closed-ended inference questions in which participants select options (Connor Desai & Reimers, 2019)

2 The additional story details in the original warehouse fire story included statements such as “Two firefighters are reported to have been taken to the hospital because of breathing toxic fumes that built up in the area in which they were working” which we replaced with “Three warehouse workers working overtime, have been taken to St. Columbus Hospital, due to smoke inhalation”. We modified these story details to avoid strongly biasing participants towards the misinformation rather than the correction. We included one statement from the original story (*“firefighters attending the scene report thick, oily smoke and sheets of flames hampering their efforts”*, so participants had the opportunity to answer the inference questions using the misinformation.

3 Details of all analyses can be found at https://osf.io/zjgx8/.

4 Examples of response coding criteria for recall questions can be found at https://osf.io/zjgx8/

5 One issue with typical CIE stimuli is that sometimes the incident described only has one potential cause presented: the corrected misinformation. It is possible that people report that cause in their inferences not because they believe it but because of pragmatic (“well it’s the only thing they mentioned so they must want me to say it”) or imaginative (“I can’t think of any other possible causes, so I’ll mention this even though I know it was corrected”) factors.

6 Some other research includes a control condition where no misinformation is presented to account for spontaneous references to the cause that is presented and corrected in the experimental conditions. The CIE can then be shown as the difference between references to corrected misinformation and spontaneous references to the same cause where misinformation is not presented. One reason for not including that here is that in these scenarios we were explicit that the corrected explanation was definitely not the cause of the event, and as such a more suitable normative baseline would be zero.
